# Supplementary material for: Gender differences in motor and non-motor symptoms in individuals with mild-moderate Parkinson’s disease
Source: PLoS One. 2023 Jan 11;18(1):e0272952. doi: 10.1371/journal.pone.0272952 (PMC9833587; doi:10.1371/journal.pone.0272952)
Supplement: Attachment — Submitted filename: Plos One R1 Abraham et al Response.docx [file pone.0272952.s002.docx]

*Dear Dr. Witt,*

*We are grateful to have the opportunity to revise this manuscript for consideration of publication in PLOS One. We have carefully considered all the reviewers’ helpful critiques and have revised the manuscript accordingly. We strongly believe the manuscript is much improved as a result. We look forward to hearing from you soon.*

*Sincerely,*

*Madeleine Hackney, Amit Abraham and co-authors.*

PONE-D-21-40790

Gender Differences in Motor and Non-Motor Symptoms in Individuals with Mild-Moderate Parkinson’s Disease

PLOS ONE

Dear Dr. Hackney,

Thank you for submitting your manuscript to PLOS ONE. After careful consideration, we feel that it has merit but does not fully meet PLOS ONE’s publication criteria as it currently stands. Therefore, we invite you to submit a revised version of the manuscript that addresses the points raised during the review process.

Please submit your revised manuscript by Apr 18 2022 11:59PM. If you will need more time than this to complete your revisions, please reply to this message or contact the journal office at [plosone@plos.org](mailto:plosone@plos.org). When you're ready to submit your revision, log on to <https://www.editorialmanager.com/pone/> and select the 'Submissions Needing Revision' folder to locate your manuscript file.

Please include the following items when submitting your revised manuscript:

- A rebuttal letter that responds to each point raised by the academic editor and reviewer(s). You should upload this letter as a separate file labeled 'Response to Reviewers'.
- A marked-up copy of your manuscript that highlights changes made to the original version. You should upload this as a separate file labeled 'Revised Manuscript with Track Changes'.
- An unmarked version of your revised paper without tracked changes. You should upload this as a separate file labeled 'Manuscript'.

If you would like to make changes to your financial disclosure, please include your updated statement in your cover letter. Guidelines for resubmitting your figure files are available below the reviewer comments at the end of this letter.

If applicable, we recommend that you deposit your laboratory protocols in protocols.io to enhance the reproducibility of your results. Protocols.io assigns your protocol its own identifier (DOI) so that it can be cited independently in the future. For instructions see: [https://journals.plos.org/plosone/s/submission-guidelines#loc-laboratory-protocols](https://nam11.safelinks.protection.outlook.com/?url=https%3A%2F%2Fjournals.plos.org%2Fplosone%2Fs%2Fsubmission-guidelines%23loc-laboratory-protocols&data=04%7C01%7Cmehackn%40emory.edu%7C8a5725a9f17c4931ffcd08d9fdb91e88%7Ce004fb9cb0a4424fbcd0322606d5df38%7C0%7C0%7C637819794628927650%7CUnknown%7CTWFpbGZsb3d8eyJWIjoiMC4wLjAwMDAiLCJQIjoiV2luMzIiLCJBTiI6Ik1haWwiLCJXVCI6Mn0%3D%7C3000&sdata=GqHy%2FvTP9VvQxOgLNWDRi9b0%2Beprv2sI8%2Fw5rmiiLtE%3D&reserved=0). Additionally, PLOS ONE offers an option for publishing peer-reviewed Lab Protocol articles, which describe protocols hosted on protocols.io. Read more information on sharing protocols at [https://plos.org/protocols?utm_medium=editorial-email&utm_source=authorletters&utm_campaign=protocols](https://nam11.safelinks.protection.outlook.com/?url=https%3A%2F%2Fplos.org%2Fprotocols%3Futm_medium%3Deditorial-email%26utm_source%3Dauthorletters%26utm_campaign%3Dprotocols&data=04%7C01%7Cmehackn%40emory.edu%7C8a5725a9f17c4931ffcd08d9fdb91e88%7Ce004fb9cb0a4424fbcd0322606d5df38%7C0%7C0%7C637819794628927650%7CUnknown%7CTWFpbGZsb3d8eyJWIjoiMC4wLjAwMDAiLCJQIjoiV2luMzIiLCJBTiI6Ik1haWwiLCJXVCI6Mn0%3D%7C3000&sdata=fQgD28I6GT780l87WLZGfLcSaZNinJ%2BC2vWZDW5%2FNgk%3D&reserved=0).

We look forward to receiving your revised manuscript.

Kind regards,

Karsten Witt
Academic Editor
PLOS ONE

Journal Requirements:

When submitting your revision, we need you to address these additional requirements.

1. Please ensure that your manuscript meets PLOS ONE's style requirements, including those for file naming. The PLOS ONE style templates can be found at

<https://journals.plos.org/plosone/s/file?id=wjVg/PLOSOne_formatting_sample_main_body.pdf> and

[https://journals.plos.org/plosone/s/file?id=ba62/PLOSOne_formatting_sample_title_authors_affiliations.pdf](https://nam11.safelinks.protection.outlook.com/?url=https%3A%2F%2Fjournals.plos.org%2Fplosone%2Fs%2Ffile%3Fid%3Dba62%2FPLOSOne_formatting_sample_title_authors_affiliations.pdf&data=04%7C01%7Cmehackn%40emory.edu%7C8a5725a9f17c4931ffcd08d9fdb91e88%7Ce004fb9cb0a4424fbcd0322606d5df38%7C0%7C0%7C637819794628927650%7CUnknown%7CTWFpbGZsb3d8eyJWIjoiMC4wLjAwMDAiLCJQIjoiV2luMzIiLCJBTiI6Ik1haWwiLCJXVCI6Mn0%3D%7C3000&sdata=Qr15sMAAP7axVZmpa8U%2BlXtb2o6F8LNu8krHisHuTU8%3D&reserved=0)

***RESPONSE***: *We have ensured that our manuscript meets PLOS ONE’s style requirements.*

2. Please amend your current ethics statement to address the following concerns:

a) Did participants provide their written or verbal informed consent to participate in this study?

*RESPONSE*: *We have clarified that all participants gave written informed consent (Methods/first paragraph).*

b) If consent was verbal, please explain i) why written consent was not obtained, ii) how you documented participant consent, and iii) whether the ethics committees/IRB approved this consent procedure.

***RESPONSE****: Consent was written and this point has been clarified in Methods.*

3. We note that the grant information you provided in the ‘Funding Information’ and ‘Financial Disclosure’ sections do not match.

***RESPONSE****: We have clarified and corroborated the information provided in Funding Information and Financial Disclosure.*

When you resubmit, please ensure that you provide the correct grant numbers for the awards you received for your study in the ‘Funding Information’ section.

***RESPONSE****: We have ensured that the correct grant numbers are provided.*

4. We note that you have indicated that data from this study are available upon request. PLOS only allows data to be available upon request if there are legal or ethical restrictions on sharing data publicly. For more information on unacceptable data access restrictions, please see <http://journals.plos.org/plosone/s/data-availability#loc-unacceptable-data-access-restrictions>.

In your revised cover letter, please address the following prompts:

a) If there are ethical or legal restrictions on sharing a de-identified data set, please explain them in detail (e.g., data contain potentially sensitive information, data are owned by a third-party organization, etc.) and who has imposed them (e.g., an ethics committee). Please also provide contact information for a data access committee, ethics committee, or other institutional body to which data requests may be sent.

b) If there are no restrictions, please upload the minimal anonymized data set necessary to replicate your study findings as either Supporting Information files or to a stable, public repository and provide us with the relevant URLs, DOIs, or accession numbers. For a list of acceptable repositories, please see <http://journals.plos.org/plosone/s/data-availability#loc-recommended-repositories>.

We will update your Data Availability statement on your behalf to reflect the information you provide.

***RESPONSE***: *Thank you, we have uploaded the minimal anonymized data set necessary to replicate our study findings in a Supplemental Section. Therefore, all data are in the manuscript and supporting files*

5. Your ethics statement should only appear in the Methods section of your manuscript. If your ethics statement is written in any section besides the Methods, please move it to the Methods section and delete it from any other section. Please ensure that your ethics statement is included in your manuscript, as the ethics statement entered into the online submission form will not be published alongside your manuscript.

***RESPONSE****:* *We have deleted the duplicative ethics statements.*

[Note: HTML markup is below. Please do not edit.]

Reviewers' comments:

Reviewer's Responses to Questions

**Comments to the Author**

**1. Is the manuscript technically sound, and do the data support the conclusions?**

**The manuscript must describe a technically sound piece of scientific research with data that supports the conclusions. Experiments must have been conducted rigorously, with appropriate controls, replication, and sample sizes. The conclusions must be drawn appropriately based on the data presented.**

Reviewer #1: Partly

Reviewer #2: Partly

2. Has the statistical analysis been performed appropriately and rigorously?

Reviewer #1: Yes

Reviewer #2: Yes

3. Have the authors made all data underlying the findings in their manuscript fully available?

The [PLOS Data policy](https://nam11.safelinks.protection.outlook.com/?url=http%3A%2F%2Fwww.plosone.org%2Fstatic%2Fpolicies.action%23sharing&data=04%7C01%7Cmehackn%40emory.edu%7C8a5725a9f17c4931ffcd08d9fdb91e88%7Ce004fb9cb0a4424fbcd0322606d5df38%7C0%7C0%7C637819794628927650%7CUnknown%7CTWFpbGZsb3d8eyJWIjoiMC4wLjAwMDAiLCJQIjoiV2luMzIiLCJBTiI6Ik1haWwiLCJXVCI6Mn0%3D%7C3000&sdata=L1w5p3BPxvdrt4zd6PmF7fuhP%2FCkmSyy64vdXs%2Fwtzo%3D&reserved=0) requires authors to make all data underlying the findings described in their manuscript fully available without restriction, with rare exception (please refer to the Data Availability Statement in the manuscript PDF file). The data should be provided as part of the manuscript or its supporting information, or deposited to a public repository. For example, in addition to summary statistics, the data points behind means, medians and variance measures should be available. If there are restrictions on publicly sharing data—e.g. participant privacy or use of data from a third party—those must be specified.

Reviewer #1: Yes

Reviewer #2: Yes

4. Is the manuscript presented in an intelligible fashion and written in standard English?

PLOS ONE does not copyedit accepted manuscripts, so the language in submitted articles must be clear, correct, and unambiguous. Any typographical or grammatical errors should be corrected at revision, so please note any specific errors here.

Reviewer #1: Yes

Reviewer #2: Yes

5. Review Comments to the Author

Please use the space provided to explain your answers to the questions above. You may also include additional comments for the author, including concerns about dual publication, research ethics, or publication ethics. (Please upload your review as an attachment if it exceeds 20,000 characters)

Reviewer #1: The authors present a cross-sectional retrospective study of gender differences in motor and non motor functions in PD patients living in the Southeast of the USA.
The novelty and relevance of their findings need to be emphasized in the discussion, with particular regard to the variables of rehabilitative interest.

***RESPONSE****:* *Thank you. We have emphasized the novelty and relevance of the findings in the Discussion, (1^st^ paragraph). The new text reads: “Novel findings of this study demonstrate for the first time that for patients from a large metro area in the southeastern US, the impact of gender is most evident in PD-specific measures of motor symptoms, QOL and activities of daily living. … Overall, the most striking differences were noted in PD-specific measures of motor and psychosocial function, while the differences between genders in non-PD specific measures were minor in this sample.”*

The paper by Iwaki et al (Mov Disord 2021, 36-106-117), that analysed gender differences in the largest sample of PD patients so far, should be discussed.

***RESPONSE****: Thank you. We have now included and addressed the paper by Iwaki et al. We referred to this paper and its findings in the Introduction (3^rd^ paragraph) and Discussion (2^nd^, 3^rd^, 6^th^ paragraphs).*

Some information on the physiopathological basis of gender differences in PD would be also advisable.

***RESPONSE****: We agree with the reviewer that presenting information on the physiopathological basis of gender differences in important. Please find a new section in the Introduction (3^rd^ and 4^th^ paragraphs) that discusses these factors in depth. Thank you for the suggestion!*

Minor comments:

Introduction, page 1: Ref 14 seems to support two opposite statements, please clarify.

***RESPONSE****: Thank you. We have corrected the sentence. The new text reads: “some studies found certain aspects (activities of daily living (ADL), cognition and communication) of PD-related QOL to be rated lesser in men,^13,14^….”*

In the methods, please clarify which are the motor-cognitive variables used.

***RESPONSE****: We have clarified the Motor Cognitive variables in Methods with a separate section.*

Reviewer #2: In this study, authors investigated clinical gender differences in a cohort of idiopathic Parkinson's disease (PD) from the southern United States. Clinical motor, cognitive and psychosocial gender differences emerged mainly favoring women as compared to men. The sample size is quite large and the clinical battery is wide including many PD-specific measures that authors described adequately in the methods. Many clinical gender differences here reported in part confirm existing literature and otherwise suggest the need of investigating sociocultural factors depending on geographic areas as important determinants of gender differences in PD. Although no biomarkers are investigated, the topic is relevant, clinically applicable, and supports personalized medicine.

***RESPONSE****: Thank you.*

The study is well conducted, thus there are only few comments with the intention to improve the paper.

***RESPONSE****: Thank you.*

1. In the introduction (page 4, lines 89-94), the authors report many factors which can explain clinical gender differences. Among them, estrogens and other reproductive factors should be mentioned with adequate references because of their important influences on dopaminergic systems and related motor and non-motor symptoms.

***RESPONSE****: We agree and have included information (see response to Reviewer 1 above) about the pathophysiological differences between genders, related to estrogens and other reproductive factors. (See Introduction (3^rd^ and 4^th^ paragraphs). We touch upon the important influence these factors have on dopaminergic systems and related motor and non-motor symptoms.*

2. Throughout all the manuscript, the authors refer to “mild-moderate PD”. However, it is not clear how they defined it since in the methods (Participants section) they reported the usual clinical criteria for PD. Despite it is not explicit, we supposed the mild-moderate definition is because of the H&Y stage. Please clarify this point.

***RESPONSE****: The reviewer is correct, Mild-moderate PD refers to stages I-III in Parkinson’s disease, and our participants were by and large representative of these stages. We have clarified this point in several locations where “mild-moderate” is iterated.*

3. The psychosocial measures here only include depression scale, other neuropsychiatric symptoms (not considered here) have been reported with gender differences in the related literature. This is a missing point that needs to be addressed in the discussion.

***RESPONSE****: We agree with the reviewer. We have added a referral to this point in the Limitations section. Added text reads: “The current study includes a single psychosocial measure of mood (BDI-II) while other neuropsychiatric symptoms (not considered in the current study) have been reported with gender differences in the related literature.”*

4. The reason to choice the number of falls in the past year as a covariate in the analyses is not clear.

***RESPONSE****: Thank you. We have added text for our reasons for including the falls in the past year as a covariate in the analyses in the Analysis section of Methods. The reason (in addition to that already given) is “*For example, falls occur more often in older women than older men in the general older adult population.”

5. According to the first comment, when authors discuss the motor gender differences that benefit women (totally aligned with the literature), they should also discuss the possible underlying mechanisms, such as the estrogens-induced neuroprotection.

***RESPONSE****: We agree and have added two substantials paragraph of text devoted to the issue of gender differences in motor performance and symptoms that benefit women and the underlying mechanisms (e.g., estrogen-induced neuroprotection) in the Introduction (See response to Reviewer 1 above) and mention it again in Discussion..*

6. In the discussion, the authors mainly compared their results to the previous evidence providing pointless details. Whereas they lack a discussion of their results in terms of biological differences, social differences, etc., a benefit from avoiding detailed comparisons with other secondary aspects and favoring a deep explanation and critical discussion is mandatory.

*RESPONSE: We appreciate the critique and have endeavored to discuss more deeply the biological and social differences that likely contributed to the observed results. We have endeavored to reduce text that contains trivial details. We discuss sociocultural factors that may have affected our cohort in the Introduction (5^th^ paragraph) and allude to it several times in the DISCUSSION.*

7. The relevance of sociocultural factors depending on geographic areas for PD gender differences is a crucial point that deserves a better discussion. The authors merely report this point as a possible explanation of findings not totally aligned with previous reports in comparable cohorts from other countries. Please provide a detailed explanation on how sociocultural and geographic factors might influence gender differen

*RESPONSE: Thank you. We agree with this important point and have included a paragraph addressing the sociocultural and geographic factors that might have influenced gender differences in this sample. The new text is found in Introduction (5^th^ paragraph) to motivate the research and this is alluded to throughout in Discussion.*

THANK YOU FOR THE HELPFUL CRITIQUES.

6. PLOS authors have the option to publish the peer review history of their article ([what does this mean?](https://nam11.safelinks.protection.outlook.com/?url=https%3A%2F%2Fjournals.plos.org%2Fplosone%2Fs%2Feditorial-and-peer-review-process%23loc-peer-review-history&data=04%7C01%7Cmehackn%40emory.edu%7C8a5725a9f17c4931ffcd08d9fdb91e88%7Ce004fb9cb0a4424fbcd0322606d5df38%7C0%7C0%7C637819794628927650%7CUnknown%7CTWFpbGZsb3d8eyJWIjoiMC4wLjAwMDAiLCJQIjoiV2luMzIiLCJBTiI6Ik1haWwiLCJXVCI6Mn0%3D%7C3000&sdata=HIUR2nSzvDqlWqDENCSpKs8H5AlDVEkhKOeT9bCQm4w%3D&reserved=0)). If published, this will include your full peer review and any attached files.


If you choose “no”, your identity will remain anonymous but your review may still be made public.


Do you want your identity to be public for this peer review? For information about this choice, including consent withdrawal, please see our [Privacy Policy](https://nam11.safelinks.protection.outlook.com/?url=https%3A%2F%2Fwww.plos.org%2Fprivacy-policy&data=04%7C01%7Cmehackn%40emory.edu%7C8a5725a9f17c4931ffcd08d9fdb91e88%7Ce004fb9cb0a4424fbcd0322606d5df38%7C0%7C0%7C637819794628927650%7CUnknown%7CTWFpbGZsb3d8eyJWIjoiMC4wLjAwMDAiLCJQIjoiV2luMzIiLCJBTiI6Ik1haWwiLCJXVCI6Mn0%3D%7C3000&sdata=IBmO3Wmzyy7TxmEnP9Gg%2BCpn6Ib%2Fu1aY8cR7j5A0Y7o%3D&reserved=0).

Reviewer #1: No

Reviewer #2: No

[NOTE: If reviewer comments were submitted as an attachment file, they will be attached to this email and accessible via the submission site. Please log into your account, locate the manuscript record, and check for the action link "View Attachments". If this link does not appear, there are no attachment files.]

While revising your submission, please upload your figure files to the Preflight Analysis and Conversion Engine (PACE) digital diagnostic tool, [https://pacev2.apexcovantage.com/](https://nam11.safelinks.protection.outlook.com/?url=https%3A%2F%2Fpacev2.apexcovantage.com%2F&data=04%7C01%7Cmehackn%40emory.edu%7C8a5725a9f17c4931ffcd08d9fdb91e88%7Ce004fb9cb0a4424fbcd0322606d5df38%7C0%7C0%7C637819794628927650%7CUnknown%7CTWFpbGZsb3d8eyJWIjoiMC4wLjAwMDAiLCJQIjoiV2luMzIiLCJBTiI6Ik1haWwiLCJXVCI6Mn0%3D%7C3000&sdata=TJFjRDPe%2F40KTlv8pRtASPCs3pzfynvWc%2FlUsrS%2B23U%3D&reserved=0). PACE helps ensure that figures meet PLOS requirements. To use PACE, you must first register as a user. Registration is free. Then, login and navigate to the UPLOAD tab, where you will find detailed instructions on how to use the tool. If you encounter any issues or have any questions when using PACE, please email PLOS at [figures@plos.org](mailto:figures@plos.org). Please note that Supporting Information files do not need this step.

*In compliance with data protection regulations, you may request that we remove your personal registration details at any time.* [*(Remove my information/details)*](https://nam11.safelinks.protection.outlook.com/?url=https%3A%2F%2Fwww.editorialmanager.com%2Fpone%2Flogin.asp%3Fa%3Dr&data=04%7C01%7Cmehackn%40emory.edu%7C8a5725a9f17c4931ffcd08d9fdb91e88%7Ce004fb9cb0a4424fbcd0322606d5df38%7C0%7C0%7C637819794628927650%7CUnknown%7CTWFpbGZsb3d8eyJWIjoiMC4wLjAwMDAiLCJQIjoiV2luMzIiLCJBTiI6Ik1haWwiLCJXVCI6Mn0%3D%7C3000&sdata=9X9Q67GUlLOTv7TeSlmkhfgB4IoR0pqTtMFmEMh3l0Q%3D&reserved=0)*. Please contact the publication office if you have any questions.*
